# High-Intensity Interval Training Versus Moderate-Intensity Continuous Training in Middle-Aged and Older Patients with Type 2 Diabetes: A Randomized Controlled Crossover Trial of the Acute Effects of Treadmill Walking on Glycemic Control

**DOI:** 10.3390/ijerph16214163

**Published:** 2019-10-28

**Authors:** Romeu Mendes, Nelson Sousa, José Luís Themudo-Barata, Victor Machado Reis

**Affiliations:** 1 EPIUnit—Instituto de Saúde Pública, Universidade do Porto, 4050-600 Porto, Portugal; 2 Northern Region Health Administration, 4000-477 Porto, Portugal; 3 Department of Sport Sciences, Exercise and Health, University of Trás-os-Montes e Alto Douro, 5000-801 Vila Real, Portugal; 4 CIDESD—Research Center in Sports Sciences, Health Sciences and Human Development, 5000-801 Vila Real, Portugal; 5 Faculty of Health Sciences, University of Beira Interior, 6200-506 Covilhã, Portugal; 6 Cova da Beira Hospital Centre, 6200-251 Covilhã, Portugal

**Keywords:** physical activity, exercise, walking, high-intensity interval training, type 2 diabetes, glycemic control, acute effects, crossover trial

## Abstract

Background: This study aimed to compare the acute effects of high-intensity interval training (HIIT) versus moderate-intensity continuous training (MICT) on glycemic control in middle-aged and older patients with type 2 diabetes (T2D), using treadmill walking as aerobic exercise mode. Methods: Fifteen patients with T2D (60.25 ± 3.14 years; glycated hemoglobin 7.03 ± 0.33%; medicated with metformin and/or gliptins), participated in a randomized controlled crossover trial. They underwent three experimental conditions (treadmill walking HIIT session (5 × (3 min at 70% of heart rate reserve (HRR) + 3 min at 30% HRR)); treadmill walking MICT session (30 min at 50% HRR); and a control session of rest (CON)) in random order and in the postprandial state. Measurements of capillary blood glucose (BG) were taken immediately before, during, and until 50 min after the experimental conditions. Results: Both HIIT and MICT treadmill walking sessions reduced BG levels during exercise and laboratory 50 min recovery period compared to CON (time*condition interaction effect; *p* < 0.001). The effect of HIIT was greater compared with MICT (*p* = 0.017). Conclusions: Treadmill walking HIIT seems a safe and more effective exercise strategy on immediate acute glycemic control compared with MICT in middle-aged and older patients with T2D under therapy with metformin and/or gliptins. Trial Registration Number: ISRCTN09240628.

## 1. Introduction

Diabetes is a global public health problem that continues to rise, currently affecting 425 million people worldwide [1]. Most diabetes cases (90% to 95%) are from type 2 diabetes (T2D) where aging and physical inactivity play major roles as risk factors [2].

Current physical activity recommendations for type 2 diabetes treatment and control [3,4] suggest a weekly accumulation of a minimum of 150 minutes of moderate-intensity aerobic exercise (40–59% of heart rate reserve (HRR) or oxygen uptake reserve (VO_2_R); or 12–13 points in a rating of perceived exertion (RPE) scale of 6 to 20 points [5]), spread over a minimum of three days per week, with no more than two consecutive days without exercise. Resistance exercise is also recommended at least two days a week (non-consecutive), as well as flexibility exercises (complementarily to other types of exercise). Alternatively, and if there are no cardiovascular or musculoskeletal contraindications, aerobic exercise dose can be accomplished by 90 min of vigorous-intensity exercise per week (60–89% of HHR or VO_2_R; or 14–17 points in a RPE scale of 6–20 points [5]).

Acute blood glucose (BG) control is crucial to reduce the risk of micro and macrovascular complications of T2D, especially in the aged individual, and exercise is one of the cornerstones of this control [6,7,8]. Although the traditional method of aerobic exercise is the moderate-intensity continuous training (MICT), between 30 to 50 min per session, exercising at higher intensities seems to offer additional benefits on glycemic control, cardiovascular risk factors, and physical fitness in patients with T2D [9,10,11]. However, performing a session of continuous vigorous-intensity exercise may pose an increased risk and discomfort and may not have applicability in T2D patients, especially in the middle-aged and older with low physical fitness, diabetes comorbidities, and higher cardiovascular risk [12,13,14].

High-intensity interval training (HIIT) has recently emerged as an attractive method to implement aerobic exercise at higher intensities even in populations with risk factors and chronic diseases, including T2D [15,16,17,18]. This exercise method is characterized by brief bouts of vigorous-intensity exercise interspersed with periods of rest or active recovery at lighter intensities [16]. This strategy allows individuals to be involved in several periods of vigorous-intensity on the same exercise session, producing a greater stimulus for cardiovascular and metabolic adaptations [19,20,21]. Nonetheless, since vigorous-exercise training presents additional contraindications and risks, namely in individuals with T2D, possible benefits of HIIT should be compared with the traditional MICT [4,22,23].

Despite the several published studies about the benefits of HIIT in patients with T2D [18,24,25,26], very few have analyzed its acute efficacy and safety on glycemic control in this population, and in direct comparison with MICT [27,28,29]. With the alarming increase in the prevalence of T2D, particularly among middle-aged and older people [1], there is a need for more effective exercise strategies to ensure the health benefits of physical activity, including metabolic control.

Hypothesizing that different training methods should have different acute metabolic effects, this study aimed to compare the acute effects of HIIT versus MICT on glycemic control in middle-aged and older patients with T2D, using treadmill walking as the aerobic exercise mode.

## 2. Materials and Methods 

### 2.1. Study Design

This was a randomized controlled crossover trial. Participants were submitted to three different experimental conditions (HIIT, MICT, and a control session of rest (CON)) in random order, with one week apart, and in the postprandial state of a standardized breakfast. 

### 2.2. Study Participants

Fifteen volunteers (eight women and seven men) were recruited from a diabetes outpatient clinic at a local hospital according to the following inclusion criteria: aged 55 to 75 years; diagnosis of T2D for at least one year; glycated hemoglobin (HbA1c) less than 10%; pharmacological regimen stabilized for at least three months (and not under insulin, insulin secretagogues, glucocorticoids, or drugs with influence on heart rate response to exercise); non-smokers in the last 6 months; major complications of diabetes screened and controlled (diabetic retinopathy, diabetic nephropathy, diabetic foot, and major cardiovascular risk factors); without limitations in gait or balance; independent living in the community; without participation in supervised exercise programs in the last 6 months; and consistent dietary pattern for at least 6 months.

Before experimental engagement, all participants underwent a detailed medical evaluation to screen for relative or absolute contraindications to vigorous-intensity exercise, including a maximal treadmill stress test to confirm the absence of underlying cardiac contraindications [4,23,30].

**Figure 1:**
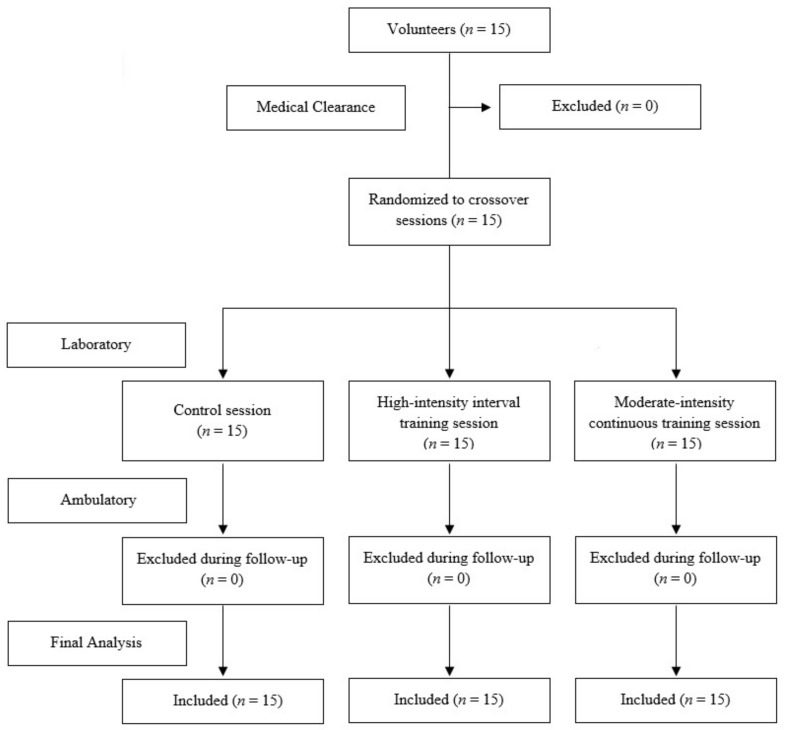
Sample flow chart.

During the study period (three weeks), the following exclusion criteria were applied: not performing all experimental conditions; not accomplishing the rules of the ambulatory period; changes in medication; changes in dietary pattern; involvement in other supervised exercise sessions; and acute illness. None of the participants were excluded from the final analysis (Figure 1).

Participants’ characteristics and pharmacological regimens are presented in Table 1.

The study’s protocol was approved by the local hospital’s ethics committee (36/2009) in accordance with the Declaration of Helsinki [31]. All individuals were informed about the risks of the research prior to signing an institutionally-approved informed consent document to participate in the study.

### 2.3. Laboratory Procedures

#### 2.3.1. Preliminary Laboratory Procedures Adaptation

One week prior to the beginning of the study, participants visited the laboratory for treadmill and food adaptation. During 15 minutes, they were trained to walk on a treadmill without hand support and to select the maximum treadmill speed without compromising gait pattern and balance. Treadmill incline was used to reach the different exercise intensities aimed to be tested in the study (moderate and vigorous). They also tasted and approved the breakfast and morning snacks that would be used in the evaluations’ visits. All laboratory and ambulatory procedures were explained. They received instructions to maintain their usual diet and not to perform exercise or strenuous physical activities in the days before the experiments. They also received information to maintain usual daily life activities during the whole study period (usual diet, habitual physical activity, and medication).

#### 2.3.2. Baseline Period

**Figure 2:**
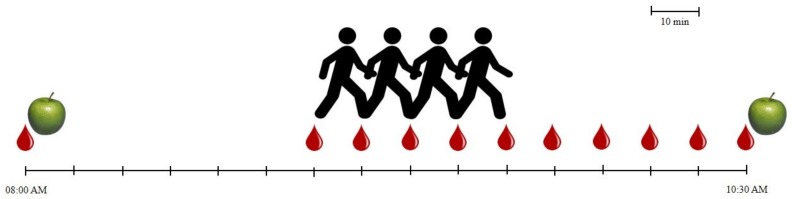
Time sequence of laboratory procedures. Drop of blood: capillary blood glucose monitoring; Apple: meal; Walking individual: exercise session.

Participants visited the laboratory for three mornings, with one week apart. They present themselves at 08:00 AM (Figure 2) with a fasting period of a minimum of 8 h. Only water was permitted at home.

Capillary blood glucose (BG) was measured through a clinically validated digital and automatic glucometer (Breeze 2, Bayer Healthcare, Mishawaka, USA [32]). Glucometer calibration was tested on each morning against a standard solution. BG was measured after capillary puncture on earlobes [33] with a specific device (Microlet 2, Bayer Healthcare, Mishawaka, USA).

After BG assessment, participants ate a standardized breakfast consisting of a low-fat drinkable yogurt (180 g), two slices of bread (50 g) with turkey ham (30 g), and water *ad libitum*. This meal provided 199.60 kcal, 30.38 g of carbohydrates, 14.80 g of proteins, 1.88 g of lipids, and 2.80 g of fiber. They also took the usual morning medications.

After breakfast participants rested for 60 minutes in seating position. During this period all laboratory and ambulatory procedures were remembered, and the morning experimental session was randomly selected by computer software (HIIT, MICT or CON). Water was available *ad libitum*.

Immediately before the experimental session start (baseline) and still in seating position, BG, blood pressure (BP), and heart rate (HR) were assessed. Blood pressure was measured using a clinically validated digital and automatic BP monitor (M6 Comfort, Omron Healthcare, Kyoto, Japan [34]) according to international recommendations [35]. HR was measured using a HR monitor with a chest band (RS800CX, Polar, Kempele, Finland). If any of these three variables was outside of the normal range of values (BG < 100 mg/dL or > 250 mg/dL; systolic BP with a ≥ 10 mmHg difference from the clinical BP values; diastolic BP with a ≥ 5 mmHg difference from the clinical BP values; and HR ≥ 100 bpm) the experimental session was cancelled and delayed for another day in order to avoid the occurrence of acute adverse events [23]. Baseline HR value was used to calculate target HR training zones using Karvonen HRR method [36].

Laboratory temperature and humidity were controlled through a digital thermo-hygrometer (KlimaLogg Pro, TFA, Wertheim, Germany), and regulated to remain around 21 °C and 50%, respectively [37].

#### 2.3.3. Exercise Protocols

HIIT session consisted of a 40 min treadmill walking session (Johnson Fitness T8000 Pro, Johnson Health Tech, Taichung, Taiwan): a 5 min warm-up at 25% of HRR, followed by 5 sets of 3 min bouts at 70% of HRR interspersed by 3 min bouts at 30% of HRR (totaling 30 min), and a 5 min cool-down period at 25% of HRR.

MICT session consisted in a 40 min treadmill walking session: a 5 min warm-up at 25% of HRR, followed by 30 minutes at 50% of HRR, and a 5 min cool-down period at 25% of HRR.

Treadmill speed and incline were adjusted in order to obtain the target HR training zones. Participants performed all exercise bouts without hand support. Treadmill speed registered in the laboratory adaptation visit was used as reference. HR was continuously recorded using an HR monitor with a chest band (RS800CX, Polar, Kempele, Finland). Borg RPE scale (6 to 20 points) was also used to monitor exercise intensity [38]. During both exercise sessions, all participants drank water *ad libitum* with a minimum ingestion of 5 mL/kg [39]. BG was measured during exercise at each 10 min (10, 20 and 30 min) and immediately at exercise ending (at 40 min).

#### 2.3.4. Control Session

During CON session, participants remained seated for 40 min. Water was available *ad libitum* and BG was assessed at 10, 20, 30 and 40 min.

#### 2.3.5. Recovery Period

After experimental conditions (HIIT, MICT and CON) participants rested on seating position during 50 min. BG was measured at each 10 min (50, 60, 70, 80 and 90 min). Feet were searched for injuries. After this 50-min recovery period and before leaving the laboratory all participants ate a snack consisting of a low-fat yogurt (125 g), three cookies (Maria-like, 18.75 g), and water *ad libitum*. This meal provided 112.19 kcal, 17.63 g of carbohydrates, 6.70 g of proteins, 1.58 g of lipids, and 0.23 g of fiber.

### 2.4. Ambulatory Procedures

After the snack, participants left the laboratory with indications to maintain normal daily life activities, usual diet, usual medication, and not to perform exercise or strenuous physical activities on that same day. They were also instructed to measure BG (with the same laboratory glucometer) immediately before each meal (lunch, afternoon snack, dinner and before bed) and on the next day at fasting state. In order to control diet, medication, and habitual physical activity each participant was asked to fill a food record template with medications included, and to wear a digital pedometer on the waist (Walking Style One HJ-152, Omron Healthcare, Kyoto, Japan) until bedtime. Other intercurrences were also asked to be registered in a formulary. On the next day participants visited the laboratory to deliver the glucometer, the pedometer, the food record, and the intercurrences formulary.

### 2.5. Statistical Analysis

Data was initially screened for normally with Shapiro-Wilk test. To compare the average of exercise intensity of HIIT and MICT (with exception of warm-up and cool-down periods) a paired samples *t*-test was used. To analyze the influence of experimental conditions on BG evolution over time a two-way (time*condition) analysis of variance (ANOVA) with repeated measures was performed. For this purpose, data was split into two different periods: 1) laboratory procedures; and 2) ambulatory follow-up. An ANOVA was conducted for each period. Partial eta squared values (*η_p_^2^*) were reported to quantify the effect sizes. To test for differences between conditions, post-hoc analysis with Bonferroni adjustments were performed. A one-way ANOVA was conducted to analyze differences on habitual physical activity (number of steps) between the ambulatory periods. The level of statistical significance was set at *p* < 0.05 and data was analyzed with IBM SPSS Statistics (version 21, New York, USA). Data is shown as mean ± standard deviation.

## 3. Results

Adherence to experimental procedures was 100%. All participants took the oral antidiabetic agents during the breakfast (metformin and/or gliptins). During experimental conditions and laboratory recovery period no exercise-related acute adverse events were recorded, such as symptomatic hypoglycemia (symptoms plus BG < 70 mg/dL [8,40]), asymptomatic level 2 hypoglycemia (BG < 54 mg/dL [8,40]), hyperglycemia (BG > 250 mg/dL [4]), foot injuries, musculoskeletal pain or discomfort, or chest angina.

On HIIT, an average intensity of 71.83 ± 2.04% of HRR was achieved during the five sets of vigorous intensity, through a walking speed of 4.21 ± 0.26 km/h and an incline of 12.83 ± 1.47%. Global exercise intensity of HIIT with recovery periods (excluding warm-up and cool-down periods) was 50.50 ± 1.93% of HRR. On MICT an average intensity of 50.25 ± 1.55% of HRR was achieved in the 30 min bout of moderate intensity through a walking speed of 4.21 ± 0.26 km/h and an incline of 6.04 ± 2.09%. No significant differences were found between global exercise intensities of both sessions (*t* = 0.338; *p* = 0.742).

Table 2. presents the values of BG at all moments of evaluation in the three experimental conditions.

**Figure 3:**
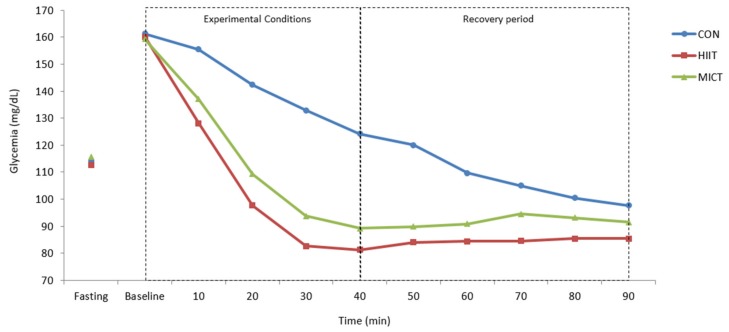
Capillary blood glucose values (mg/dL) during laboratory procedures: fasting state; immediately before (baseline), during (10, 20 and 30 min), and immediately after the experimental conditions (40 min); and during recovery periods (50, 60, 70, 80 and 90 min). CON: control; HIIT: high intensity interval training; MICT: moderate intensity continuous training. A significant time*condition interaction effect (two-way ANOVA with repeated measures) was identified for BG values evolution (*F* = 11.783; *p* < 0.001; *η_p_^2^* = 0.517). Significant differences were observed (post-hoc analysis with Bonferroni adjustments) between HIIT and CON (*p* < 0.001), between MICT and CON (*p* < 0.001), and between HIIT and MICT (*p* = 0.017).

A significant time*condition interaction effect was identified for BG values evolution in laboratory (Figure 3; *F* = 11.783; *p* < 0.001; *η_p_^2^* = 0.517). Significant differences were observed between HIIT and CON (*p* < 0.001), between MICT and CON (*p* < 0.001), and between HIIT and MICT (*p* = 0.017).

During the ambulatory follow-up period no intercurrences were registered. All participants did the programmed meals (lunch, afternoon snack, dinner, and before bed) approximately at the same schedules and without qualitative changes on their food content. Medication was always the same and on the same schedules. Ambulatory habitual physical activity was not significantly different between the three experimental conditions (CON 7896.75 ± 2191.03 steps vs. HIIT 7320.83 ± 2245.69 steps vs. MICT 7386.08 ± 1875.62 steps; *F* = 2.146; *p* = 0.141; *η_p_^2^* = 0.163).

**Figure 4:**
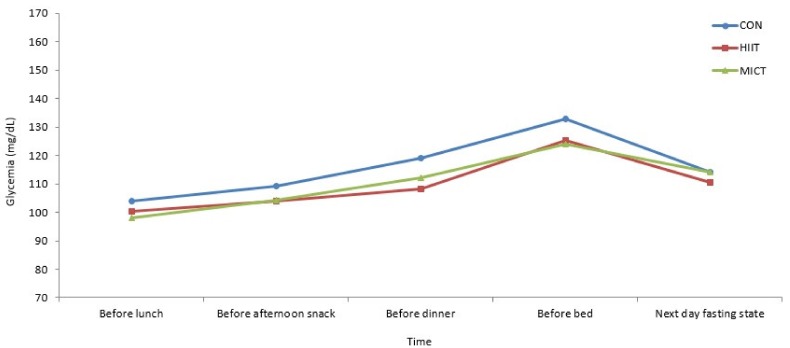
Capillary blood glucose values (mg/dL) during ambulatory follow-up periods: before each meal and next day fasting state. CON: control; HIIT: high intensity interval training; MICT: moderate intensity continuous training. No significant time*condition interaction effect (two-way ANOVA with repeated measures) was identified for BG values evolution (*F* = 0.348; *p* = 0.944; *η_p_^2^* = 0.031).

No significant time*condition interaction effect was identified for BG values evolution in the ambulatory follow-up period (Figure 4; *F* = 0.348; *p* = 0.944; *η_p_^2^* = 0.031).

## 4. Discussion

The main finding of this study is that a session of treadmill walking HIIT reduced BG at a greater extent compared to MICT (duration- and intensity-matched) in middle-aged and older patients with T2D (under pharmacological therapy with metformin and/or gliptins), and without acute adverse effects. Although both aerobic exercise training sessions had a superior influence on acute glycemic control compared to CON, the effect of HIIT was the best, at least during exercise and a 50 min recovery period. 

HIIT has recently assumed a prominent role in the scope of physical activity and health due to the cardiovascular and metabolic benefits that it appears to induce in populations with risk factors and chronic diseases, including T2D [15,16,17,18].

Our study confirms these expectations in middle-aged and older patients with T2D, in direct comparison to the traditional MICT, and with an exercise protocol that can be easily replicated in facilities with treadmills such as Sports and Fitness Clubs, and Rehabilitation Centres. Since vigorous-intensity exercise has several contraindications and risks in individuals with T2D [23], it is only relevant to recommend HIIT if benefits are greater compared to MICT.

Despite that we found in the scientific literature several studies that aimed to analyze the acute effects of aerobic HIIT in T2D, only very few compared the benefits of this novel exercise method on glycemic control with MICT, although with different methodologies [27,28,29].

Karstoft, et al. [29] compared the effects of two duration- and intensity-matched treadmill walking protocols: HIIT (10 × (3 min at 89% of VO_2_peak [peak oxygen uptake for walking ~ 81% participants’ VO_2_max] + 3 min at 54% of VO_2_peak)) vs. MICT (60 min at 73% of VO_2_peak), on 10 patients with T2D (60.3 ± 2.3 years; randomized controlled crossover design). They analyzed the impact on postprandial glycemic control (four-hour mixed meal tolerance test) and free-living glycemic control (continuous glucose monitoring). The results showed that HIIT was significantly better compared with MICT on both outcomes.

Terada, et. al. [27] developed a randomized controlled crossover trial with patients with T2D (n = 10; 60 ± 6 years) to study the effects of HIIT vs. MICT (duration- and intensity-matched protocols on treadmill walking) on 24 h continuous glucose monitoring. HIIT protocol was 15 × (1 min at 100% VO_2_peak [~ participants’ VO_2_max] + 3 min at 40% of VO_2_peak). MICT protocol was 60 min at 55% of VO_2_peak. HIIT significantly reduced nocturnal and fasting glycemia on the day following exercise, with a greater reduction compared to MICT.

In another study, and a few years earlier, Terada, et al. [28] compared the effects of HIIT and MICT in a more simple design, through the analysis of pre- and post-exercise capillary BG levels of 703 exercise sessions from a supervised exercise program with both protocols (matched for frequency, duration and intensity; 12 weeks; 5 sessions per week of 30–60 min of treadmill walking alternated with stationary cycling;). Patients with T2D (n = 15, 55–75 years) were randomized to HIIT (sets with 1 min at 100% VO_2_R + 3 min at 20% VO_2_R) or MICT (40% VO_2_R) programs. HIIT was a significant predictor of glucose-lowering effect of exercise, although pre-exercise BG levels were the strongest predictor.

In order to compare both aerobic training methods our HIIT and MICT protocols were also designed to have the same duration and to be intensity-matched (same global average exercise intensity—50% of HRR). We used a HIIT protocol with 5 sets of 3 min bouts at 70% HHR, interspersed with 3 min bouts of active recovery at 30% of HRR—a 3:3 ratio (same as in Karstoft et al. [29]) allowing a good recovery of participants between sets. The MICT protocol tested the traditional exercise recommendation for T2D control—30 min of moderate-intensity aerobic exercise on most days of the week in order to accumulate a minimum of 150 min per week [3,4]. We added to both protocols warm-up and cool-down periods in order to prevent injuries and adverse events.

Walking on a treadmill was also our exercise mode. On the preliminary visit to the laboratory, participants selected the maximum treadmill speed without hand support and without compromising gait pattern and balance (4.0 to 4.5 km/h). This allowed for achieving the target intensities with manipulation of incline rather than speed. Higher speeds could compromise data collection.

Research with walking protocols can have a greater translation into clinical practice, since walking is the most popular aerobic exercise mode for public health promotion and for T2D control [3,41]. Walking is a low-cost, low-impact, and low-risk activity that can be practiced outdoors with few resources and with an acute metabolic effect on glycemic control [42,43]. However, brisk walking is an activity typically of moderate intensity [44] and we had to manipulate treadmill incline to high values in every participant (10% to 15%) to achieve vigorous intensity. This type of HIIT protocol can be replicated in outdoor conditions if walking is combined with stair climbing, uphill walking, walking with external loads, or with very brisk walking [44,45,46].

There is an unlimited number of possible HIIT protocols using different exercise modes, intensities, number of sets, and interval lengths which are difficult direct comparisons between studies [24].

The results of our study were obtained with strict control in laboratory conditions of the variables that could interfere with the BG levels, such as food intake, physical activity, and medication. Laboratory visits on fasting state and standardized breakfast were determinant.

The baseline period allowed participants to begin the three experimental conditions on a homogeneous metabolic state (with a minimum average difference of 2 mg/dL on BG). The recovery period was crucial to control post-exercise BG response.

It was in laboratory conditions that our main results were highlighted. After the 40 min session of HIIT, BG dropped about 42 mg/dL compared to CON, and about 9 mg/dL compared to MICT.

After the recovery period (50 min), this difference was attenuated to 11 mg/dL and 7 mg/dL compared with CON and MICT, respectively. This attenuation was important to prevent hypoglycemia. The physiological protection mechanisms against hypoglycemia, such as the consumption of muscle and hepatic glycogen stores, and the production of glucose from other energy substrates, could have contributed to this response [47].

Mechanisms mediating the greater reduction in glycemia with HITT cannot be ascertained from this study. However, HIIT imposes an increased cardiovascular and neuromuscular stimulus in comparison with MICT, namely a recruitment of a larger proportion of muscle fibers. The underlying molecular mechanisms seem to be related to increased activation of peroxisome proliferator-activated receptor gamma coactivator 1-alpha (PGC-1α), which is a mediator of the expression of several mitochondrial genes, leading to a greater capacity for glucose uptake and oxidation, and enhanced insulin sensitivity [15,16,20,21].

It is important to underline that the CON session also significantly reduced BG levels (a mean reduction of 37 mg/dL from baseline after 40 min, and 64 mg/dL after 90 min). This reduction on BG levels is naturally related with time but also with the synergistic effect of the oral hypoglycemic agents for glycemic control taken at breakfast [48,49,50]. This fact highlights the importance of having a control session in this type of studies aiming to analyze the acute effects of exercise on clinical conditions and under pharmacological therapy. Karstoft, et al. [29] and Terada, et al. [27] also included a CON session of seated rest to compare results.

Safety issues also play a major role on analyzing HIIT effects. The inclusion criteria of our participants, and the detailed medical evaluation, allowed the application of a vigorous-intensity exercise protocol without any recorded symptomatic exercise-related acute adverse event in laboratory, nor any intercurrence during ambulatory follow-up period. Medical clearance including a cardiological stress test is recommended for patients with T2D that aim to engage in vigorous-intensity exercise even without cardiovascular disease symptoms [4,23,30]. However, medical evaluations and stress tests are financially and logistically costly and may represent an additional important barrier to exercise practice in this population [4,51]. Similar studies also included this type of pre-exercise health screening and evaluation to guarantee safety [27,28,29].

Although exercise may increase risk of hypoglycemia, only the individuals under insulin or insulin secretagogues (sulfonylureas and meglitinides) therapy seem to be at risk during, immediately after, or several hours after exercise. Exercise-related hypoglycemia is rare in patients medicated with other types of oral antidiabetic drugs such as metformin and gliptins [6,23,47]—medications taken by our participants. To minimize the risk of hypoglycemia, some studies suspended antidiabetic drugs before and during the days of the experiment [27,29]. These studies had individuals under therapies of different combinations of sulfonylureas, metformin, and gliptins, but no insulin therapy.

The option by a sub-maximal exercise protocol (70% of HHR), the adequate warm-up and cool-down periods, the care with hydration during exercise, and the standardized meals (before and after exercise) also contributed to the safety of our study. All exercise sessions were monitored by a clinical exercise physiologist with experience in emergency procedures, including basic life support [37].

The significant results observed in laboratory settings were not registered in the ambulatory follow-up period. There were no significant differences between the three experimental conditions (HIIT, MICT and CON). Studies of Terada, et al. [27] and Karstoft, et al. [29] observed significant benefits in glycemic control in the follow-period of the exercise protocol with HIIT, but with the use of continuous glucose monitoring. This is the preferred method to detect acute ambulatory changes in glycemic control [52,53].

During the ambulatory period, we tried to control some important confounding factors such as food intake, physical activity, and medication. After leaving the laboratory, all participants had instructions to maintain normal daily life activities, usual diet, usual pharmacological treatment, and not to perform exercise or strenuous physical activities on that same day. During this period physical activity was monitored by a digital pedometer like in Terada, et al. study [27]. This equipment seems valid to monitor habitual physical activity in patients with T2D [54,55] and in the elderly [56,57], although without the possibility to assess physical activity intensity. This issue could be assessed with triaxial accelerometers such as the ones used by Karstoft, et al [29].

Ambulatory dietary patterns were not quantitatively assessed due to the lack of standardization of food and beverages portions sizes; this process required a specific training of the patients. However, food records were analyzed by a dietitian in order to assess qualitative changes in meals and their schedules. Similar studies conducted a quantitative analysis of food records [27,29]. To highlight the importance of monitoring food intake, and its relation with exercise practice, a study conducted by Dube, et al. [58] in patients with diabetes revealed that appetite sensations and food consumption are greater after exercise sessions with higher reductions in BG levels.

It would also be important to perform a quantitative assessment of 24-hour pre-experimental conditions of physical activity and dietary pattern.

Despite the above-mentioned limitations, our study is strengthened by the randomized crossover design; the control session without exercise to assess the effect of time and pharmacological therapy; the baseline conditions starting on the fasting state; the standardized breakfast and morning snack; the use of a popular and easy-to-implement exercise mode; and the follow-up ambulatory period until the next morning with control of food intake and physical activity.

The results of this study have potential implications for physical activity promotion for T2D control in middle-aged an older patients. Our data can help exercise professionals evaluate the advantages and disadvantages of HIIT and to prescribe it through a safe and effective protocol for acute glycemic control, and preferably integrate it into a long-term regular exercise program.

Few studies have already observed significant greater benefits of HIIT on physical fitness, glycemic control, body composition, and insulin sensitivity in patients with T2D that underwent aerobic exercise programs of HIIT, compared to MICT [13,45,59,60,61]. Some included treadmill [13,61] and outdoor walking protocols [45,59]. However, the results are still inconsistent.

The ideal HIIT protocol for T2D control is far from being established, and more research is needed especially in the direct comparison of different protocols.

## 5. Conclusions

Treadmill walking HIIT seems a more effective exercise strategy for immediate acute glycemic control compared to MICT in middle-aged and older patients with T2D. This exercise method appears to be safe in T2D patients with pre-exercise clinical evaluation, and under pharmacological therapy with metformin and/or gliptins.

## Figures and Tables

**Table 1:** Participants’ characteristics and pharmacological regimen.

Variable	Mean ± Standard Deviation
Age (years)	60.25 ± 3.14
Diabetes duration (years)	5.33 ± 2.31
Glycated hemoglobin (%)	7.03 ± 0.33
Clinical systolic blood pressure (mmHg)	123.33 ± 10.47
Clinical diastolic blood pressure (mmHg)	74.25 ± 8.13
Body mass index (kg/m^2^)	29.57 ± 4.61
Oral antidiabetic agents	n = 15 (100.00%)
Metformin only	n = 6 (40.00%)
Metformin + Sitagliptin	n = 5 (33.33%)
Metformin + Vildagliptin	n = 4 (26.67%)

**Table 2:** Mean values (± standard deviation) of capillary blood glucose (mg/dL) at all moments of evaluation in the three experimental conditions.

Time	CON	HIIT	MICT
Fasting state	114.25 ± 24.65	112.67 ± 21.98	115.75 ± 21.84
Baseline	161.25 ± 26.89	160.17 ± 30.90	159.25 ± 24.62
10 min	155.50 ± 33.38	128.08 ± 29.36	137.00 ± 32.99
20 min	142.42 ± 31.62	97.75 ± 25.55	109.25 ± 27.56
30 min	132.92 ± 31.43	82.75 ± 21.65	93.75 ± 25.90
40 min	124.17 ± 29.94	81.33 ± 18.00	89.25 ± 20.82
50 min	120.08 ± 29.54	84.08 ± 14.43	89.92 ± 15.07
60 min	109.75 ± 26.54	84.50 ± 11.00	90.92 ± 16.17
70 min	105.00 ± 25.86	84.58 ± 9.89	94.58 ± 14.96
80 min	100.42 ± 22.98	85.42 ± 9.78	93.17 ± 14.94
90 min	97.75 ± 25.06	85.50 ± 11.01	91.50 ± 14.52
Before lunch	104.00 ± 28.19	100.42 ± 15.36	98.00 ± 11.95
Before afternoon snack	109.17 ± 28.60	103.92 ± 19.25	104.42 ± 23.13
Before dinner	119.00 ± 19.48	108.17 ± 14.08	112.08 ± 24.95
Before bed	132.92 ± 39.35	125.33 ± 26.17	123.83 ± 39.19
Next day fasting state	114.08 ± 24.27	110.50 ± 17.73	114.00 ± 22.31

CON: control; HIIT: high intensity interval training; MICT: moderate intensity continuous training.
